# Clinicopathological characteristics of breast cancer patients from Northern Tanzania: common aspects of late stage presentation and triple negative breast cancer

**DOI:** 10.3332/ecancer.2021.1282

**Published:** 2021-09-07

**Authors:** Marianne Gnanamuttupulle, Oliver Henke, Shilanaiman Hilary Ntundu, Furaha Serventi, Leila E Mwakipunda, Patrick Amsi, Alex Mremi, Kondo Chilonga, David Msuya, Samuel G Chugulu

**Affiliations:** 1 Department of General Surgery, Kilimanjaro Christian Medical Centre, PO Box 3010, Moshi, Tanzania; 2 Faculty of Medicine, Kilimanjaro Christian Medical University College, PO Box 2240, Moshi, Tanzania; 3 Cancer Care Centre, Kilimanjaro Christian Medical Centre, PO Box 3010, Moshi, Tanzania; 4 Department of Pathology, Kilimanjaro Christian Medical Centre, PO Box 3010, Moshi, Tanzania

**Keywords:** immunohistochemistry patterns, staging, breast cancer, Tanzania, Africa

## Abstract

**Purpose:**

Breast cancer (BC) is the second most common cancer among Tanzanian women. Oestrogen (ER), progesterone and human epidermal growth factor receptor 2 play major roles in prognosis and treatment but data for Tanzania are sparse. This study aimed to determine these patterns and histological types, tumour grading and staging of BC patients in northern Tanzania for a better understanding of BC in the Sub-Saharan African (SSA) setting.

**Methods:**

A cross-sectional study recorded newly diagnosed BC cases at Kilimanjaro Christian Medical Centre between October 2018 and March 2019. Receptor status, histological types and grade, clinical stage and socio-demographic were recorded and descriptive and bivariate analyses performed.

**Results:**

116 patients were enrolled. Median age was 53 years, 71.6% were ≥45 years. The commonest molecular subtype was triple negative breast cancer (TNBC) (*n* = 33; 28.4%). One hundred and two (87.9%) patients had invasive ductal carcinoma (IDC), poorly differentiated tumours (60; 51.7%) and clinical stage III disease (62; 53.0%). ER negative tumours were associated with poorly differentiated histological grade (relative risk (RR): 1.34 (0.87–2.07)), tumour size > 5 cm (RR: 1.67 (0.33–8.35)) and IDC (RR: 3.35 (0.56–20.23)). Clinical stages III & IV (odds ratio (OR): 1.64 (0.63–4.24)) were associated with hormone receptor (HR) negative tumours and metastasis (OR: 1.60 (0.68–3.74)) with TNBC. 18% of the patients reported about first-degree relatives with BC.

**Conclusions:**

Most patients presented in advanced stages and TNBC in their menopause. HR negative tumours were associated with poor histological differentiation and IDC. The high percentage of positive family history of BC and the differences in receptor patterns compared to other parts of the world should urge further genetic research on BC in SSA.

## Background

The rising cancer burden among men and women has accounted to 18.1 million new cases and 9.6 million cancer related deaths in the year 2018 [1]. Globally, one in six women develops cancer in their life time and one out of 11 women die from the disease [1]. Breast cancer (BC) is the leading cancer type, in both incidence and mortality among women worldwide [1]. Among 8.6 million newly diagnosed cancers in women, BC accounted for 2.1 million (24.2%) and among 4.2 million cancer related deaths in women, BC caused 630,000 (15.0%) in 2018 [1]. Globally, a striking disparity in survival rates is observed among women with BC: Higher survival rates are seen among women in developed countries in contrast to the developing countries [2]. Within Africa, North African women had a higher BC incidence (29.3/100,000) than Sub-Saharan African (SSA) women (22.4/100,000) [3]. Africa and Asia have higher proportions of cancer related mortality (7.3% and 57.3%) than incidence (5.8% and 48.4%) and is attributed by higher frequencies of cancers associated with poorer prognosis, with limited access to health care, and late-stage presentation in many countries in these regions [1]. In Tanzania, with respect to estimates from the Global Cancer Observatory (GLOBOCAN) in 2018, cancer incidence in women was 25,028 of which 3,037 (12.1%) attributed to BC while the cancer mortality in women was 16,501 with 1,303 (7.9%) deaths from BC [4]. A projected 82% increase in BC incidence by 2030 has been predicted in Tanzania [5] due to changes in reproductive factors, implementation of screening programmes for prevention and early detection and improvement in health facilities [6].

Characterisation and classification of BC hormone receptor (HR) status oestrogen (ER), progesterone (PR) and human epidermal growth factor receptor 2 (HER2) are of utmost importance to determine the type of treatment and prognosis. These biomarkers in BC have been widely studied and documented in developed countries. In SSA, the patterns of these receptors are not well understood and studies reported from different countries in SSA show wide variations [7]. Triple negative breast cancer (TNBC) was the most common molecular subtype among west and central African patients [8], while hormonal receptor positive tumours were common in east African countries [9]. Eng *et al* [10] reported a wide range (40%–80%) of ER positive BC in North African women and a range of 20%–70% of ER positivity West African women, which points to a wider variation among indigenous Africans. Moreover, studies report that BC in SSA and African Americans is more aggressive than in Caucasians and African patients present with a higher clinical stage and younger age as well as hormonal receptor negative disease [8, 11–15]. Given the geographical diversity of the molecular subtypes of BC in the African continent, these studies cannot generalise a higher aggressiveness of BC between the African populations and their Caucasian counterparts.

In Tanzania, few data about receptor status in BC patients exist and they have been published from two sites in the western and eastern regions of Tanzania. This study aimed to close the knowledge gap by focusing on the population of the north-east region. We determined receptor patterns, histopathological types, grade and clinical stage of BC in women attending Kilimanjaro Christian Medical Centre (KCMC), the referral and zonal hospital of north-eastern Tanzania.

## Methods

### Study design and setting

A cross-sectional study was performed from beginning of October 2018 to end of March 2019 at KCMC. This hospital has a capacity of 500 beds and belongs to the four consultant hospitals in the country. Its catchment hosts a population of approximately 12 million people and extends into parts of the neighbouring country Kenya. The hospital has a general surgery department with a capacity of 35 beds. The department has four consultants in general surgery, the pathology department has two pathologists and the cancer care centre (oncology department) is managed by two oncologists. This cancer care centre provides chemotherapy services and patients are referred to the National Cancer Institute in the commercial city (Dar-es-Salaam) for radiation services.

### Study participants

All female patients confirmed to have BC by histopathological diagnosis at the pathology department following routine processing who self-presented or were referred to the General Surgery department (in- and out-patient) during the study period were included.

Patients with BC diagnosed from cytological smears only, patients with metastatic cancer from other sites to the breast, male patients and patients who had their biopsy procedures in other health facilities were excluded.

Demographic and clinicopathological characteristics of patients were recorded from the medical records. Tumour grade was assessed by using Nottingham Combined Histologic Grade [16]. Assessment of tumour size, nodal involvement and metastasis status was based on TNM classification [17]. HR status ER, PR and HER2 expressions were determined using standard immunohistochemistry (IHC) protocol.

### Ethical considerations

Ethical approval was granted by Research Ethical Committee of Kilimanjaro Christian Medical University College of Tumaini University. The standards of good clinical practice and good research practice have been maintained according to the Helsinki declaration.

### Formalin fixed paraffin embedded block preparation

All specimens were obtained through an incision or core needle biopsy and immediately fixed in 10% buffered formalin in less than 5 minutes. These breast tissue blocks which were confirmed by routine haematoxylin and eosin staining for primary histological diagnosis of invasive BC were taken for IHC staining.

The formalin-fixed, paraffin-embedded breast tumour blocks were cut into 3 μm thick sections for each block and then deparaffinised by placing them on a 60°C hot plate for 15–30 minutes. Xylene (two changers) was used to dewax and rehydration was done using alcohol grades of 100%, 90% and 70%, respectively. Antigen retrieval was performed by using a pressure cooker for 40 minutes. Then the slides were cooled by using cold distilled water followed by placing the slides in a citrate buffer for 5 minutes. Tris Buffered Saline was used to wash the slides and then the endogenous activity was blocked using 3% hydrogen peroxide in Phosphate Buffered Saline. The primary antibodies (ER (clone EP1) and ready to use dilution; PR (clone 636) and ready to use dilution; HER2 protein (clone HercepTest) and ready to use dilution)) provided by DAKO supplies Ltd. were used and incubated for 30 minutes and were followed by Horseradish peroxidase secondary antibody. Finally, diaminobenzidine was used to visualise the staining and the slides were counter stained with haematoxylin.

### Immunohistochemistry

Preparation and fixation of the samples were performed as per the American Society of Clinical Oncology/College of American Pathologists (ASCO/CAP) guidelines [18]. Receptor testing of ER, PR, HER2, tumour grade and histological types was analysed before initiation of chemotherapy and fixed within 24 hours to minimise antigen degradation. Tissue sections of 3 µm were H&E stained, checked for fixation quality and invasive cell adequacy. Immunostaining for ER, PR and HER2 was performed manually.

HER2 receptor staining intensity was determined by the protein expression on tumour cell membrane and scored as 0, 1+, 2+ and 3+ (ASCO/CAP, HER2 Guidelines, 2018), and ≤1+ was considered HER2 negative, 2+ was considered HER2 equivocal and 3+ was considered HER2 positive [19]. Fluorescent in situ hybridisation (FISH) was not performed for HER2 equivocal 2+ results because of unavailability. Therefore, HER2 equivocal results in this study were considered as positive.

ER and PR were considered positive if ≥1% nuclei of tumour cells were stained according to ASCO/CAP guidelines [20].

BC molecular subtypes were determined using the IHC markers as follows: Luminal A (positive ER and/or PR with negative HER2), Luminal B (positive ER and/or PR with positive HER2), HER2 enriched (negative ER and PR with positive HER2) and triple negative (TNBC) (ER, PR and HER2 negative) [21].

### Data analysis

Statistical data analysis was performed using SPSS software version 25.0 (IBM SPSS statistics for Windows NY; IBM Corp). For categorical variables, data were summarised in proportions and frequency tables. For continuous variables, median and inter-quartile range (IQR) were used to summarise data.

The *p*-values for categorical variables were calculated for IHC subtypes and demographic and clinical variables using chi-square (*X*^2^). Logistic regression was used to determine odds ratio (OR) and relative risk (RR).

## Results

Final analysis included 116 patients and 28 patients were excluded (6 male patients, 13 biopsies were not obtained at KCMC and in 9 patients, no immunostaining was performed due to logistic constraints).

Table 1 displays the demographics of all participants as well as the clinical characteristics.

Sixty-two (53.4%) patients were negative for ER and 88 (75.9%) patients were negative for PR. 44% of patients were HER2 negative (0 and 1+) and the same number of patients was HER2 positive (3+). Equivocal results were seen in 14 (12%) patients (2+). Among the concordant 84 (72.4%) and discordant 32 (27.5%) patients, hormonal receptor positive and negative tumours were 57 (49.1%) and 59 (50.9%), respectively. The molecular subtype categorisation was as follows: TNBC 33 (28.4%), Luminal B 30 (25.9%), Luminal A 28 (24.1%) and HER2 enriched 25 (21.6%) (Table 2).

### Molecular subtype by age group at diagnosis

**Figure 1.**
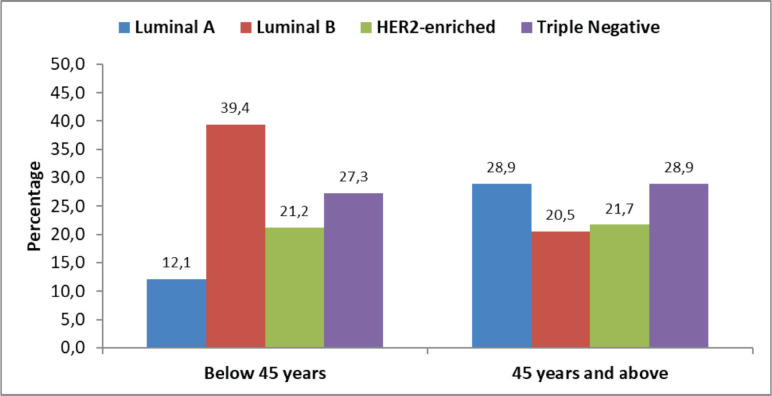
Molecular subtypes by age at diagnosis (*n* = 116).

Thirty-three (28.4%) patients were below 45 years and 83 (71.6%) patients were 45 years and above at the time of diagnosis. The majority of the patients below 45 years were diagnosed with Luminal B molecular subtype (*n* = 13; 39.4%) patients each, while TNBC and Luminal A were the commonest molecular subtype in patients who were ≥45 years (*n* = 24; 28.9%). The least common subtype seen in patients aged <45 years was Luminal A (*n* = 4; 12.1%) patients, while Luminal B tumours were the least common subtype in patients ≥45 years (*n* = 17; 20.5%). HER2 enriched tumours were equally balanced in both groups with 7 patients (21.2%) in the younger and 18 patients (21.7%) in the older group (Figure 1).

### Association of clinicopathological parameters with HR negative and TNBC

Both variables ‘clinical stage III/IV’ and ‘tumour size > 5 cm’ were associated with higher odds of being ER and PR negative tumours (see Table 3) while the presence of distant metastasis had two times higher odds of TNBC. None of these correlations reached statistically significant levels (Table 3).

### Binomial logistic regression analysis for the RR ratio of ER negative BC; stratified by age, tumour size, histological grade, lymph node involvement (LNI) and histological type

On binomial logistic regression, poorly differentiated histological grade tumours had 1.3 times higher risk of being ER negative tumours (RR (95% CI): 1.34 (0.87–2.07), *p* = 0.19) while invasive ductal carcinoma (IDC) had three times higher risk of being ER negative tumours (RR (95% CI): 3.35 (0.56–20.23), *p* = 0.19). Tumours > 5 cms in size had two times higher risk of being ER negative tumours (RR (95% CI): 1.67 (0.33–8.35), *p* = 0.53] (Table 4).

### Binomial logistic regression analysis for RR ratio of ER and PR negative and TNBC stratified by age, tumour size, histological grade, LNI and histological type

Poorly differentiated histological grade tumours had 1.4 times higher risk of being ER and PR negative tumours while IDC had three times higher risk of being ER and PR negative tumours. Tumours more than 5 cms in size had two times higher risk of being ER and PR negative tumours. Furthermore, poorly differentiated tumours had two times higher risk of being TNBC and IDC had a two times higher risk of being TNBC (Table 5).

### Histological types and grade

One hundred and two (87.9%) patients were diagnosed with IDC and 6 (5.2%) patients had invasive lobular carcinoma (ILC). Poorly differentiated tumours were common in 60 (51.7%) patients compared to well differentiated tumours in 13 (11.2%) patients.

### Clinical stage

Sixty-two (53.0%) patients were diagnosed in stage III, 32 (28.0%) in stage IV, 20 (17.0%) in stage II and only 2 (2.0%) patients in stage I.

## Discussion

Though several studies on BC have been published in SSA, there are only a few studies conducted in Tanzania and for the northern part of the country almost no data exist. Thus, this study was conducted to address the clinicopathological findings of patients in this region. The major findings in regard to the IHC pattern were that half of the patients had HR positive tumours while TNBC was reported in 28.4% followed by the HER2 enriched tumours as the least common molecular subtype. The majority of the patients were diagnosed with IDC and half of the patients had poorly differentiated tumours. 81% of the patients had an advanced stage disease (stage III or IV) at diagnosis.

### Age and menopause

The most commonly affected age group was 45 years and above with a mean age of 53 years. Our findings are similar to the other studies done in Tanzania by Mwakigonja* et al* [22] and in Nigeria by Adesunkanmi *et al* [23], who reported the mean age at diagnosis as 49 years and 48 years, respectively. African British women too present with a similar mean age of 46 years [24], but this age was lower than their Caucasian counterparts as revealed in studies done in Europe and America [25, 26].

BC seems to affect younger individuals in Africa and of African descent living in other regions of the world [27, 28]. Therefore, BC poses a higher negative economic burden to affected families in Africa.

Almost 70% of our patients were postmenopausal, which is conclusive with studies conducted in Europe [29–31] but stands in contrast with a Ugandan study that displayed 69% of premenopausal women with BC [28]. This shows – again – the differences among the African population but bearing in mind that most of the studies are single centre studies with small number of participants.

### Family history

About one third of the participants reported a history of BC or cervical cancer in the family, and 18.1% of these patients reported to have a first-degree relative diagnosed to have BC. In a study reported by Ford *et al* [32], families with BRCA1 and BRCA2 mutations had 28% and 37% of women with BC, respectively. As these germ line mutations were not analysed, we cannot draw conclusion on these genetic mutations in our study but the high percentage of familiar BC suggests genetic mutations as common.

### Late stage presentation

Most of our patients had a tumour size at diagnosis of ≥5 cm and only 2.6% had tumours less than 2 cm. Similar findings were reported by other studies in Tanzania [22, 27] and Nigeria [33]. Studies from India and Italy reported that the majority of their patients had smaller tumours with less than 2 cm and 2–5 cm and only few patients presented with bigger tumours [34, 35]. Furthermore, almost 80% had lymph node metastasis at the time of diagnosis, which corresponds to other publications from Tanzania, Kenya and India [9, 27, 34]. A quarter of our participants presented with distant metastasis. In contrast, the ‘Carolina BC Study’ in the USA found that majority of the patients 61% did not have distant or lymph node metastasis at the time of diagnosis [36]. This might be a feature of either more aggressive BC and/or late stage presentation in indigenous Africans than in their Caucasian counterparts, driven by poor health knowledge, financial constrains and both limited health facilities and screening programmes [22, 27, 37].

### Hormone receptor expression

In contrast to studies conducted earlier in Kenya [9] and the USA [36] that displayed 71% and 60% HR positive patients, our study cohort showed less than 50% positivity. Several other studies performed on African descendents reported as well lower ER rates but these studies were based mainly on archive samples with the risk of biological degradation of the samples and false negative results. This can be excluded from our study and our findings are in line with another study from Tanzania [22] and from Sudan and Eritrea [15], showing also less than half of the patients HR positive. In conclusion, HR positive among Africans remains highly heterogeneous among different regions, but it can be said to be in general lower than in Caucasians.

Positive PR expression was lower in our cohort and in a study conducted previously in Tanzania [22]. But again, the previously mentioned studies from Sudan and Eritrea (47.7%) [15] are also in the PR expression in line with our findings. That stands in stark contrast to findings from Caucasian populations with a generally higher expression [21].

### HER2 receptor

Considering equivocal results as positive, our cohort shows almost half of all participants positive for HER2, while two other studies from Tanzania reported positive HER2 expression on BC to be only 15% [22] and 26% [38]. Neither of these studies reported equivocal results. As mentioned earlier, FISH could not be performed, but if it is performed on equivocal results, approximately 21% of the samples will be interpreted as positive [39]. It is likely that we overestimated the positive HER2 results, but we would still have 44% positive results, considering equivocal results as negative. Low HER2 positive results were not only found in other Tanzanian studies but also noted in the Eritrean and Sudanese cohort [15] and among British black BC patients [24].

### TNBC

About one third of the patients had TNBC. Other studies reported from regions with African descendents presented similar results; 38.4% [27], 29.2% [33] and 36.0% [40]. Mwakigonja *et al* [22] reported a higher proportion 45.6% of patients with TNBC, but with a small sample size of only 46 patients. The ‘Carolina BC Study’ reported a significant higher prevalence of TNBC (26%) in African Americans than their non-African American counterparts (16%) [36] which is conclusive with studies from China, Northern America and India (11.1% [41], 13.4% [21] and 16.7% [42]).

These results on BC HRs demonstrate genetic multiplicity of BC across races and among different groups in indigenous Africans in SSA but bearing in mind the non-representativeness of most of the SSA studies.

In our study on molecular subtypes, the biggest difference between younger and older patients can be found in Luminal A and B. While in the young cohort <45 years of age, Luminal B was more prevalent with 40%, it was only found in 29% in patients aged ≥45 years and Luminal A tumours were correspondingly 10% lower in the younger group. These differences can be attributed to higher proportions of HER2 positive BC in our study as discussed above.

### Histological grading

Our patients with ER negative BC and TNBC were more likely to have poorly differentiated histological grade tumours which are in line with previous findings [7, 43]. This implies that aggressive grades of BC are mostly seen in ER negative and TNBC patients. A previous study under the cooperation of KCMC’s pathology department displayed the same result with a higher proportion of high grade tumours compared to white American women with BC [44]. While these findings are associated with shorter survival and distant recurrence of the tumour [45], the aforementioned study also assessed tumour infiltrating lymphocytes (TILs). TILs are known to correlate with a better treatment outcome and prognosis in patients from European ancestry [44] and interestingly, the study revealed higher proportions of TIL involvement in the Tanzanian patients compared to white American BC patients. Stratified to ER negative patients only, the difference was not significant and further studies are needed to understand this correlation and which influence infections and other environmental exposures might have on the anti-tumour immune response [44]. A Japanese study has shown that high TIL grades within the ER negative BC patients predict better relapse-free survival and cancer-specific survival, while it is a poor prognostic marker for ER positive patients [46]. Whether the high ER negative proportion among the Tanzanian BC patients in combination with high TIL grades leads to good therapy outcomes remains to be proofed by prospective studies.

The most common histological type of BC in this study was IDC and was reported in 87.9% patients. Also, this finding corresponds to studies in Africa and Europe [22, 47]. Majority of patients in a study conducted in the USA reported features of IDC in 72.7% patients [21]. This indicates that IDC is the most common histological type across many races and regions.

### Clinical staging

Stage III BC is the most common stage at diagnosis followed by stage IV, together accounted to more than 80%. Similar trend was reported in Uganda by Galukande *et al* [48], in their study. An opposite trend was reported in a study performed in four European countries and Australia whereby patients with BC in stages I and II were the vast majority [49]. This shows that patients in our region have an advanced disease at the time of diagnosis compared to Caucasian patients or patients from developed regions of the world. It should prompt action on health education and promotion of screening programmes in Northern Tanzania as this has been proven to be effective for earlier presentation of BC patients [50].

Clinical stage III tumours were the majority in all four molecular subtype groups. Majority of patients with HER2-enriched molecular subtype tumours had stage III tumours. A previous study has reported that women with HER2 enriched molecular subtype tumours exhibit 26% higher risk of mortality within 5 years of diagnosis and higher mortality was associated with the stage at diagnosis and the HER2 receptor status [51]. This demonstrates the aggressiveness of HER2-enriched tumours among our study participants.

### Limitations

FISH was not performed to evaluate HER2+ equivocal results due to unavailability. Also, the proliferative index marker, Ki67 was not performed which could have contributed to a better understanding of the aggressiveness of the tumour. Because of the limited number of participants, the results should be interpreted with caution and cannot be generalised.

## Conclusions

The data presented in this study suggest that BC in Northern Tanzania is more aggressive and is of a higher stage at diagnosis compared to Western countries. In general, most of the data corresponds with other studies from SSA and contributes to the body of evidence about the different biology of BC in this part of the world. However, the remarkable different results on HR and HER2 demonstrate the genetic multiplicity of BC among different groups in African descendents. Even though interpretation must be done cautiously as most of the studies from SSA are not representative samples of the population, but the heterogeneous results should promote more genetic studies in the future to understand the real world setting in SSA. Enhancing health literacy and expanding prevention programmes should be a focus of health policy makers in Tanzania to reduce the high rate of late presentation in BC.

## Declaration

### Ethics approval and consent to participate

This study was approved by Research Ethical Committee of Kilimanjaro Christian Medical University College of Tumaini University with a certificate No.2354, and permission was granted to access patient’s information. Consent to participate was not applicable.

### Consent for publication

Not applicable.

### Availability of data and material

Data will be available upon contacting corresponding author on reasonable request.

### Competing interests

All authors have no conflicts of interests.

### Funding

There was no specific funding received for this research.

### Authors’ contributions

MG: study design, data analysis, interpretation, drafting and critical review of manuscript. DM, FS, PA, AM and KC: critical review of manuscript. LEM: data collection and critical review of manuscript. OH: statistical analysis, data interpretation, drafting and critical review of manuscript. SHN: study design, statistical analysis, data interpretation, drafting and critical review of manuscript. SGC: supervision, study design, statistical analysis, data interpretation, drafting and critical review of manuscript.

## Figures and Tables

**Table 1.** Socio-demographic and clinical characteristics of participants (*N* = 116).

Variables	*n*	%
**Age years (Median, IQR)**	53 (43–65)	
**Age group (years)**		
<45	33	28.4
≥45	83	71.6
**Marital status**		
Married	89	76.7
Single	15	12.9
Divorce	5	4.3
Widow	7	6.1
**Level of education**		
Primary	61	52.6
Secondary	12	10.3
Higher	32	27.6
No education	11	9.5
**Residence**		
Rural	96	82.7
Urban	20	17.3
**Occupation**		
Farmer	53	45.7
Small-scale business	32	27.6
Professional	27	23.3
Housewife	4	3.4
**Insurance coverage**		
Yes	76	65.5
No	40	34.5
**Alcohol intake**		
Yes	78	67.2
No	38	32.8
**History of cancer in the family**		
History of BC	27	23.3
First degree	21	18.1
Second degree	6	5.2
No history of BC	56	48.3
Unknown	33	28.4
History of cervical cancer	5	4.3
**Menopausal status**		
Premenopausal	37	31.9
Postmenopausal	79	68.1
**Contraceptive use**		
Yes	78	67.2
No	38	32.8
**Type of contraceptives (*n* = 78)**		
Hormonal contraceptives	71	91.0
Other contraceptives	7	9.0
**Parity**		
Nulliparous	6	5.2
1–2	31	26.7
≥3	79	68.1
**Age at pregnancy (years) (*n* = 110)**		
<20	20	18.2
20–29	87	79.1
≥30	3	2.7
**Tumour size (cms)**		
<2	3	2.6
2–5	32	27.6
>5	81	69.8
**LNI**		
Absent	27	23.3
Present	89	76.7
**Distant metastasis**		
Absent	79	68.1
Present	37	31.9
**Site of metastasis (*n* = 37)**		
Lung	16	43.2
Spine	9	24.3
Both lung and spine	4	10.8
Others (liver, brain & contralateral breast)	8	21.6

**Table 2.** Proportions of BC hormone (ER & PR) and HER2 receptors (*n* = 116).

Hormonal receptor status	Status	*n*	%
**ER**	Positive	54	46.6
	Negative	62	53.4
**PR**	Positive	28	24.1
	Negative	88	75.9
**HER2**	Positive (3+)	51	44.0
	Negative (0 and 1+)	51	44.0
	Equivocal (2+)	14	12.0
**Concordant and discordant**			
**^a^Concordant (*n* = 84)**	ER+PR+	25	29.8
	ER−PR−	59	70.2
**^b^Discordant (*n* = 32)**	ER+PR−	29	90.6
	ER−PR+	3	9.4
**Hormonal receptor positive tumours**	ER+PR+	57	49.1
**Hormonal receptor negative tumours**	ER−PR−	59	50.9
**Molecular subtype**	Luminal A	28	24.1
	Luminal B	30	25.9
	HER2-enriched	25	21.6
	Triple negative	33	28.4

a Both ER and PR either positive or negative

b ER is positive or negative and PR is negative or positive, respectively

**Table 3.** Association of clinicopathological parameters with HR negative and TNBC in study participants (*n* = 116).

Clinical and pathological tumour characteristics	*N*	Statistical estimate for the hormonal receptor status					
		ER & PR negative			Triple negative		
		*n* (%)	OR (95% CI)	*p*-value	*n* (%)	OR (95% CI)	*p*-value
**Age (years)**							
<45	33	16 (48.5)	0.88 (0.39–1.97)	0.75	9 (27.3)	0.92 (0.37–2.28)	0.86
≥45	83	43 (51.8)	ref		24 (28.9)	ref	
**Tumour size (cm)**							
<2	3	1 (33.3)	ref		1 (33.3)	ref	
2–5	32	14 (43.8)	1.56 (0.12–19.74)	0.73	7 (21.9)	0.56 (0.04–7.44)	0.66
>5	81	44 (54.3)	2.38 (0.20–27.90)	0.48	25 (30.9)	0.89 (0.08–10.46)	0.93
**Histological grade**							
Poorly	60	30 (50.0)	0.44 (0.12–1.64)	0.21	14 (23.3)	0.36 (0.10–1.27)	0.10
Moderately	43	20 (46.5)	0.39 (0.10–1.51)	0.15	13 (30.2)	0.51 (0.14–1.84)	0.29
Well	13	9 (69.2)	ref		6 (46.2)	ref	
**LNI**							
Absent	27	12 (44.4)	ref		9 (33.3)	ref	
Present	89	47 (52.8)	1.40 (0.59–3.34)	0.44	24 (27.0)	0.74 (0.29–1.88)	0.52
**Metastasis**							
Absent	79	40 (50.6)	ref		20 (25.3)	ref	
Present	37	19 (51.4)	1.03 (0.47–2.26)	0.94	13 (35.1)	1.60 (0.68–3.74)	0.28
**Clinical stage**							
Early (I & II)	22	9 (40.9)	ref		6 (27.3)	ref	
Late (III & IV)	94	50 (53.2)	1.64 (0.63–4.24)	0.30	27 (28.7)	1.08 (0.38–3.05)	0.89

LNI, Lymph node involvement

**Table 4.** Binomial logistic regression analysis for RR ratio of ER negative BC stratified by age, tumour size, histological grade, LNI and histological type (*n* = 116).

Variable	Categories	Risk ratio	95% CI	*p*-value
Age group (years)	<45	ref		
	≥45	1.14	0.77–1.71	0.51
				
Tumour size (cms)	<2	ref		
	2–5	1.5	0.29–7.71	0.63
	>5	1.67	0.33–8.35	0.53
				
Histological grade	Well differentiated	ref		
	Moderately differentiated	0.10	0.68–1.45	0.96
	Poorly differentiated	1.34	0.87–2.07	0.19
				
LNI	Absent	ref		
	Present	1.04	0.69–1.57	0.85
				
Histological type	ILC (NST)	ref		
	IDC	3.35	0.56–20.23	0.19
	Others^a^	3.00	0.44–20.44	0.26

LNI, Lymph node involvement; IDC (NST), Invasive ductal carcinoma (no special type); ILC, Invasive lobular carcinoma

a Invasive adenosquamous cell carcinoma, Paget’s disease and anaplastic BC

**Table 5.** Binomial logistic regression analysis for RR ratio of ER and PR negative and TNBC stratified by age, tumour size, histological grade, LNI and histological type (*n* = 116).

Variable	Categories	ER & PR negative		TNBC	
		RR (95% CI)	*p*-value	RR (95% CI)	*p*-value
**Age group (years)**	<45	ref			
	≥45	1.07 (0.71–1.61)	0.75	1.06 (0.55–2.03)	0.86
					
**Tumour size (cm)**	<2	ref			
	2–5	1.31 (0.25–6.82)	0.75	0.66 (0.12–3.70)	0.63
	>5	1.63 (0.32–8.17)	0.55	0.92 (0.18–4.74)	0.93
					
**Histological grade**	Well differentiated	ref			
	Moderately differentiated	0.93 (0.61–1.40)	0.73	1.30 (0.68–2.47)	0.43
	Poorly differentiated	1.38 (0.90–2.15)	0.15	1.98 (0.94–4.17)	0.07
					
**LNI**	Absent	ref			
	Present	1.19 (0.75–1.90)	0.73	0.81 (0.43–1.52)	0.51
					
**Histological type**	ILC (NST)	ref			
	IDC	3.18 (0.53–19.19)	0.21	1.76 (0.29–10.83)	0.54
	Others^a^	3.00 (0.44–20.44)	0.26	1.50 (0.17–12.94)	0.71

LNI, Lymph node involvement; IDC (NST), Invasive ductal carcinoma (no special type); ILC, Invasive lobular carcinoma

a Invasive adenosquamous cell carcinoma, Paget’s disease and anaplastic BC
